# Disodium hydrogen citrate sesquihydrate, Na_2_HC_6_H_5_O_7_(H_2_O)_1.5_

**DOI:** 10.1107/S2056989016009014

**Published:** 2016-06-14

**Authors:** Alagappa Rammohan, Amy A. Sarjeant, James A. Kaduk

**Affiliations:** a Atlantic International University, Honolulu HI , USA; b Department of Chemistry, Northwestern University, Evanston IL, USA; c Illinois Institute of Technology, Department of Chemistry, 3101 S. Dearborn St., Chicago IL 60616, USA

**Keywords:** crystal structure, density functional theory, citrate, sodium

## Abstract

The crystal structure of disodium hydrogen citrate sesquihydrate, 2Na_2_^+^·C_6_H_6_O_7_^2−^·1.5H_2_O, has been solved and refined using laboratory X-ray single-crystal diffraction data, and optimized using density functional techniques. The asymmetric unit contains two independent hydrogen citrate anions, four sodium cations and three water molecules. The coordination polyhedra of the cations (three with a coordination number of six, one with seven) share edges to form isolated 8-rings. The un-ionized terminal carb­oxy­lic acid groups form very strong hydrogen bonds to non-coordinating O atoms, with O⋯O distances of 2.46 Å.

## Abstract 2

The crystal structure of disodium hydrogen citrate sesquihydrate has been solved and refined using laboratory X-ray single-crystal diffraction data, and optimized using density functional techniques.

## Chemical context   

In the course of a systematic study of the crystal structures of Group 1 (alkali metal) citrate salts to understand the anion’s conformational flexibility, ionization, coordination tendencies, and hydrogen bonding, we have determined several new crystal structures. Most of the new structures were solved using powder diffraction data (laboratory and/or synchrotron), but single crystals were used where available. The general trends and conclusions about the 16 new compounds and 12 previously characterized structures are being reported separately (Rammohan & Kaduk, 2016*a* ▸). Four of the new structures – NaKHC_6_H_5_O_7_, NaK_2_C_6_H_5_O_7_, Na_3_C_6_H_5_O_7_, and a second polymorph of NaH_2_C_6_H_5_O_7_ – have been published recently (Rammohan & Kaduk, 2016*b* ▸,*c* ▸,*d* ▸,*e* ▸) and two additional structures – KH_2_C_6_H_5_O_7_ and KH_2_C_6_H_5_O_7_(H_2_O)_2_ – have been communicated to the CSD (Kaduk & Stern, 2016*a* ▸,*b* ▸).
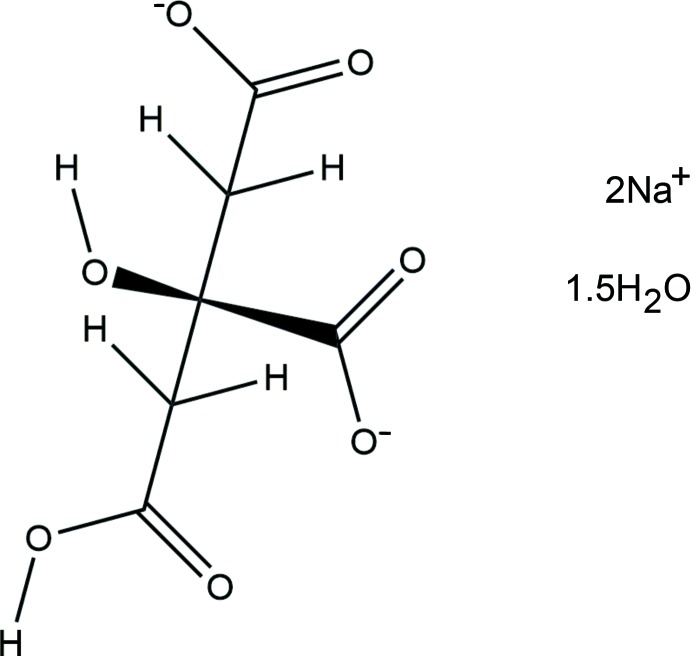


## Structural commentary   

**Figure 1:**
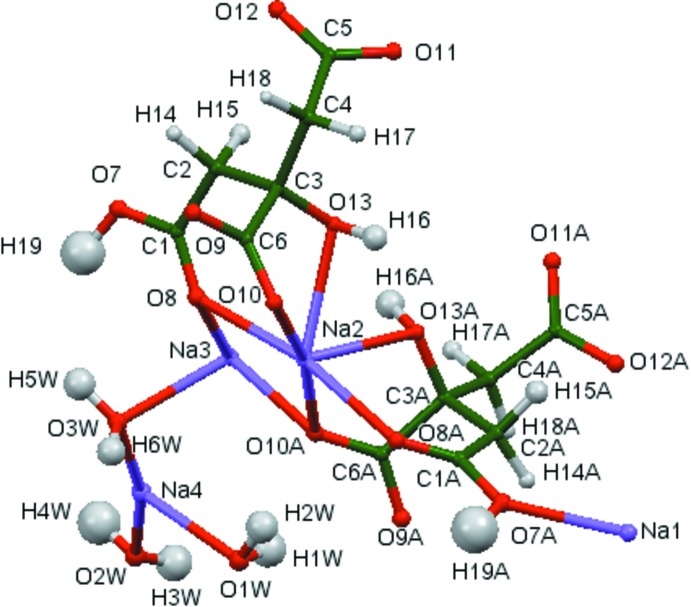
The asymmetric unit of the DFT-optimized structure, with the atom numbering. The atoms are represented by 50% probability spheroids.

**Figure 2:**
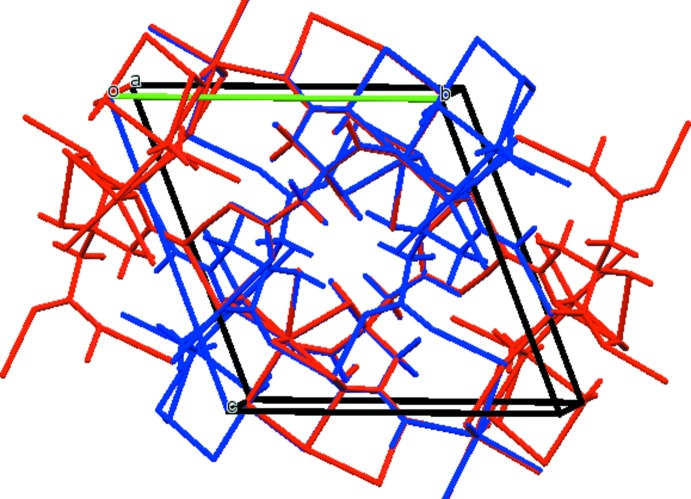
Comparison of the refined and optimized structures of disodium hydrogen citrate sesquihydrate. The refined structure is in red, and the DFT-optimized structure is in blue.

The asymmetric unit of the title compound is shown in Fig. 1 ▸. The root-mean-square deviation of the non-hydrogen atoms in the refined and DFT-optimized structures is only 0.048 Å. The excellent agreement between the two structures (Fig. 2 ▸) is strong evidence that the experimental structure is correct (van de Streek & Neumann, 2014 ▸). This discussion uses the DFT-optimized structure. Almost all of the bond lengths, bond angles, and torsion angles fall within the normal ranges indicated by a *Mercury Mogul* geometry check (Macrae *et al.*, 2008 ▸). Only the C3—O13 bond length [observed = 1.416 (2), optimized = 1.410, *Mogul* average = 1.445 (11) Å, Z-score = 3.3] and the C2—C3—C4—C5 torsion angle [observed = −55.7 (1), optimized = −50.6°] are flagged as unusual. The standard deviation on the *Mogul* average for the C3—O13 distance is exceptionally low, resulting in the elevated Z-score. The C2—C3—C4—C5 torsion angle lies in the tail of a minority gauche conformation. None of the experimental qu­anti­ties are flagged as unusual.

The two independent citrate ions in the optimized structure are very similar; the root-mean-square displacement of the non-hydrogen atoms is 0.10 Å. Both anions occur in a *gauche,trans* conformation, which is one of the two low-energy conformations of an isolated citrate. The central carboxyl­ate and hydroxyl groups are in the normal planar arrangement. The central and one terminal carboxyl­ate groups in each hydrogen citrate anion are deprotonated. Both citrates chelate to Na2 atom through the end carboxyl­ate atom O8, the central carboxyl­ate atom O10, and the hydroxyl group O13.

The four independent Na1, Na2, Na3, and Na4 cations are 6-, 7-, 6-, and 6-coordinate. The 6-coordinate Na^+^ cations are in an approximately octa­hedral environment. The bond-valence sums are 1.12, 1.26, 1.16, and 1.20, respectively. Only the oxygen atoms O12 and O12*A* do not coordinate to an Na atom; these are part of central carboxyl­ate groups, and the Na—O distances are very long at 2.76 Å. There are one, one, one, and three water mol­ecules in the coordination spheres of atoms Na1, Na2, Na3, and Na4.

## Supra­molecular features   

**Figure 3:**
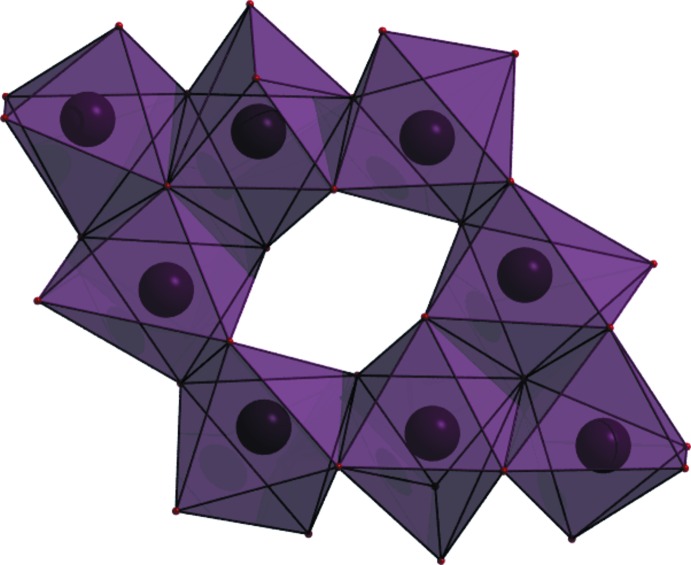
The 8-rings formed by edge sharing of the Na coordination polyhedra.

**Figure 4:**
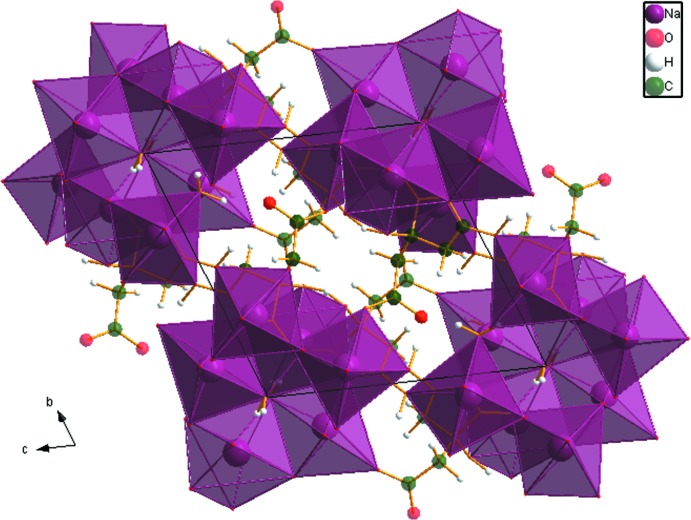
The crystal structure of Na_2_HC_6_H_5_O_7_(H_2_O)_1.5_, viewed down the *a* axis.

The [NaO_*x*_ coordination polyhedra (*x* = 6, 7) share edges to form 8-ring units (Fig. 3 ▸), which are isolated from each other in the crystal structure (Fig. 4 ▸).

The OH functions of the carboxy groups, O7—H19 and O17*A*—H19*A*, form very strong hydrogen bonds to the non-coordinating atoms O12*A* and O12, respectively (Table 1 ▸). The experimental donor–hydrogen distances are significantly longer than the DFT-optimized ones. The refined O7—H19 and O7*A*—H19*A* distances are both 1.20 (3) Å, and the optimized distances are both 1.079 Å. The other hydrogen bonds participate in a variety of rings.

## Database survey   

Details of the comprehensive literature search for citrate structures are presented in Rammohan & Kaduk (2016*a* ▸). The observed powder pattern matched that of Na_2_HC_6_H_5_O_7_(H_2_O)_2_ in PDF entry 00-016-1182 (de Wolff *et al.*, 1966 ▸) A reduced-cell search in the Cambridge Structural Database (Groom *et al.*, 2016 ▸) yielded 104 hits, but limiting the chemistry to C, H, Na, and O only resulted in no hits.

## Synthesis and crystallization   

The sample was purchased from Sigma–Aldrich (lot #BCBC6031V). Single crystals were isolated from the as-received material.

## Refinement details   

Crystal data, data collection and structure refinement details are summarized in Table 2 ▸. All hydrogen-atom parameters were refined.

## DFT Calculations   

A density functional geometry optimization (fixed experimental unit cell) was carried out using *CRYSTAL09* (Dovesi *et al.*, 2005 ▸). The basis sets for the C, H, and O atoms were those of Gatti *et al.* (1994 ▸), and the basis set for Na was that of Dovesi *et al.* (1991 ▸). The calculation used 8 *k*-points and the B3LYP functional, and took about 10 days on a 2.4 GHz PC. *U*_iso_ values were assigned to the optimized fractional coordinates based on the *U*_eq_ values from the refined structure.

## Supplementary Material

Crystal structure: contains datablock(s) na2c, na2c_DFT. DOI: 10.1107/S2056989016009014/vn2112sup1.cif

Structure factors: contains datablock(s) na2c. DOI: 10.1107/S2056989016009014/vn2112na2csup2.hkl

Structure factors: contains datablock(s) na2c_DFT. DOI: 10.1107/S2056989016009014/vn2112na2c_DFTsup3.hkl

CCDC references: 1483449, 1483448

Additional supporting information:  crystallographic information; 3D view; checkCIF report

## Figures and Tables

**Table 1:** Hydrogen-bond geometry (Å, °) [Chem scheme1]

*D*—H⋯*A*	*D*—H	H⋯*A*	*D*⋯*A*	*D*—H⋯*A*
O7*A*—H19*A*⋯O12	1.079	1.393	2.465	171.1
O7—H19⋯O12*A*	1.079	1.382	2.456	172.5
O13*A*—H16*A*⋯O11*A*	0.986	1.725	2.698	168.3
O13—H16⋯O11	0.987	1.760	2.743	173.4
O1*W*—H1*W*⋯O10	0.988	1.806	2.772	165.0
O3*W*—H5*W*⋯O12*A*	0.981	1.751	2.714	165.9
O3*W*—H6*W*⋯O9*A*	0.979	1.945	2.881	159.0
O1*W*—H2*W*⋯O10*A*	0.980	2.122	3.067	161.4
O2*W*—H4*W*⋯O12	0.971	2.171	2.877	128.5
O2*W*—H3*W*⋯O8	0.972	2.146	2.946	138.6
O2*W*—H3*W*⋯O1*W*	0.972	2.503	3.166	125.3

**Table 2:** Experimental details

Crystal data	
Chemical formula	2Na_2_^+^·C_6_H_6_O_7_^2−^·1.5H_2_O
*M*_r_	263.11
Crystal system, space group	Triclinic, *P* 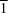
Temperature (K)	100
*a*, *b*, *c* (Å)	8.6713 (3), 10.6475 (4), 10.9961 (4)
α, β, γ (°)	68.461 (1), 79.617 (2), 81.799 (2)
*V* (Å^3^)	925.63 (6)
*Z*	4
Radiation type	Cu *K*α
μ (mm^−1^)	2.34
Crystal size (mm)	0.24 × 0.14 × 0.06

Data collection	
Diffractometer	Bruker Kappa *APEX* CCD area detector
Absorption correction	Multi-scan (*SADABS*; Bruker, 2006 ▸)
*T*_min_, *T*_max_	0.652, 0.753
No. of measured, independent and observed [*I* > 2σ(*I*)] reflections	9177, 3235, 3137
*R*_int_	0.021
(sin θ/λ)_max_ (Å^−1^)	0.599

Refinement	
*R*[*F* ^2^ > 2σ(*F* ^2^)], *wR*(*F* ^2^), *S*	0.026, 0.070, 1.10
No. of reflections	3235
No. of parameters	370
H-atom treatment	All H-atom parameters refined
Δρ_max_, Δρ_min_ (e Å^−3^)	0.36, −0.31

The same symmetry and lattice parameters were used for the DFT calculation. Computer programs: *APEX2* and *SAINT* (Bruker, 2006 ▸), *XM* and *XL* (Bruker, 2004 ▸), *OLEX2* (Dolomanov *et al.*, 2009 ▸), *DIAMOND* (Brandenburg, 2006 ▸) and *Materials Studio* (Dassault Systemes, 2014 ▸).

## supplementary crystallographic information

### (na2c) Disodium hydrogen citrate sesquihydrate Crystal data

**Table:**

2Na^+^·C_6_H_6_O_7_^2^^−^·1.5H_2_O	*Z* = 4
*M**_r_* = 263.11	*F*(000) = 540
Triclinic, *P*1	*D*_x_ = 1.888 Mg m^−^^3^
*a* = 8.6713 (3) Å	Cu *K*α radiation, λ = 1.54184 Å
*b* = 10.6475 (4) Å	Cell parameters from 7113 reflections
*c* = 10.9961 (4) Å	θ = 4.4–67.1°
α = 68.461 (1)°	µ = 2.34 mm^−^^1^
β = 79.617 (2)°	*T* = 100 K
γ = 81.799 (2)°	Rod, colourless
*V* = 925.63 (6) Å^3^	0.24 × 0.14 × 0.06 mm

### (na2c) Disodium hydrogen citrate sesquihydrate Data collection

**Table:**

Bruker Kappa APEX CCD area detector diffractometer	3235 independent reflections
Radiation source: microsource	3137 reflections with *I* > 2σ(*I*)
MX optics monochromator	*R*_int_ = 0.021
Detector resolution: 8 pixels mm^-1^	θ_max_ = 67.6°, θ_min_ = 4.4°
ω and φ scans	*h* = −7→10
Absorption correction: multi-scan (*SADABS*; Bruker, 2006)	*k* = −12→12
*T*_min_ = 0.652, *T*_max_ = 0.753	*l* = −13→12
9177 measured reflections	

### (na2c) Disodium hydrogen citrate sesquihydrate Refinement

**Table:**

Refinement on *F*^2^	Primary atom site location: dual
Least-squares matrix: full	Secondary atom site location: difference Fourier map
*R*[*F*^2^ > 2σ(*F*^2^)] = 0.026	Hydrogen site location: inferred from neighbouring sites
*wR*(*F*^2^) = 0.070	All H-atom parameters refined
*S* = 1.10	*w* = 1/[σ^2^(*F*_o_^2^) + (0.0351*P*)^2^ + 0.4756*P*] where *P* = (*F*_o_^2^ + 2*F*_c_^2^)/3
3235 reflections	(Δ/σ)_max_ = 0.002
370 parameters	Δρ_max_ = 0.36 e Å^−^^3^
0 restraints	Δρ_min_ = −0.31 e Å^−^^3^

### (na2c) Disodium hydrogen citrate sesquihydrate Special details

**Table:**

Experimental. *SADABS* (Bruker,2006) was used for absorption correction. R(int) was 0.0787 before and 0.0318 after correction. The Ratio of minimum to maximum transmission is 0.8655. The λ/2 correction factor is 0.0015.
Geometry. All esds (except the esd in the dihedral angle between two l.s. planes) are estimated using the full covariance matrix. The cell esds are taken into account individually in the estimation of esds in distances, angles and torsion angles; correlations between esds in cell parameters are only used when they are defined by crystal symmetry. An approximate (isotropic) treatment of cell esds is used for estimating esds involving l.s. planes.
Refinement. Refinement of F^2^ against ALL reflections. The weighted R-factor wR and goodness of fit S are based on F^2^, conventional R-factors R are based on F, with F set to zero for negative F^2^. The threshold expression of F^2^ > 2sigma(F^2^) is used only for calculating R-factors(gt) etc. and is not relevant to the choice of reflections for refinement. R-factors based on F^2^ are statistically about twice as large as those based on F, and R- factors based on ALL data will be even larger.

### (na2c) Disodium hydrogen citrate sesquihydrate Fractional atomic coordinates and isotropic or equivalent isotropic displacement parameters (Å^2^)

**Table:**

	*x*	*y*	*z*	*U*_iso_*/*U*_eq_	
Na1	−0.36905 (6)	0.67174 (5)	1.09406 (5)	0.01145 (13)	
Na2	−0.26692 (6)	0.20337 (5)	0.79550 (5)	0.01118 (14)	
Na3	0.08801 (6)	1.08405 (5)	0.67885 (5)	0.01112 (13)	
Na4	0.34928 (6)	0.86038 (5)	0.85665 (5)	0.01460 (14)	
O1W	0.26747 (14)	0.99605 (11)	0.99770 (10)	0.0155 (2)	
H1W	0.333 (3)	1.053 (3)	0.943 (3)	0.047 (7)*	
H2W	0.181 (3)	1.034 (2)	0.983 (2)	0.037 (6)*	
O2W	0.62348 (12)	0.82465 (11)	0.87230 (11)	0.0161 (2)	
H3W	0.679 (3)	0.886 (3)	0.849 (3)	0.047 (7)*	
H4W	0.647 (4)	0.789 (3)	0.814 (3)	0.067 (9)*	
O3W	0.09169 (12)	0.84523 (10)	0.82906 (11)	0.0134 (2)	
H5W	0.065 (3)	0.813 (2)	0.779 (2)	0.036 (6)*	
H6W	0.029 (3)	0.813 (2)	0.901 (2)	0.029 (5)*	
O7	−0.16831 (11)	0.95459 (10)	0.52380 (9)	0.0127 (2)	
O8	−0.20033 (11)	1.04882 (10)	0.67830 (9)	0.0123 (2)	
O9	−0.63550 (11)	1.06594 (9)	0.68976 (9)	0.0109 (2)	
O10	−0.53711 (11)	1.17365 (9)	0.79545 (9)	0.0107 (2)	
O11	−0.46736 (11)	1.50841 (9)	0.30164 (9)	0.0108 (2)	
O12	−0.49461 (11)	1.33279 (9)	0.24551 (9)	0.0114 (2)	
O13	−0.36210 (11)	1.32227 (10)	0.57450 (9)	0.0098 (2)	
H16	−0.419 (2)	1.378 (2)	0.603 (2)	0.023 (5)*	
C1	−0.23366 (15)	1.04395 (13)	0.57558 (13)	0.0091 (3)	
C2	−0.35405 (16)	1.14161 (13)	0.49599 (13)	0.0092 (3)	
H14	−0.413 (2)	1.0882 (17)	0.4733 (16)	0.009 (4)*	
H15	−0.291 (2)	1.1960 (18)	0.4148 (18)	0.015 (4)*	
C3	−0.46014 (15)	1.23514 (13)	0.55909 (13)	0.0087 (3)	
C4	−0.58579 (15)	1.31651 (13)	0.46879 (13)	0.0094 (3)	
H17	−0.648 (2)	1.3845 (17)	0.5050 (17)	0.011 (4)*	
H18	−0.6557 (19)	1.2561 (16)	0.4648 (15)	0.006 (4)*	
C5	−0.51150 (15)	1.39448 (13)	0.33037 (13)	0.0090 (3)	
C6	−0.55179 (15)	1.15103 (13)	0.69346 (13)	0.0087 (3)	
O7A	−0.33313 (12)	0.47238 (10)	1.04866 (9)	0.0137 (2)	
O8A	−0.32692 (11)	0.32406 (9)	0.94823 (9)	0.0112 (2)	
O9A	0.10082 (11)	0.29981 (10)	0.93250 (9)	0.0125 (2)	
O10A	0.00229 (11)	0.19514 (9)	0.82508 (10)	0.0118 (2)	
O11A	−0.01900 (11)	0.69477 (9)	0.48444 (9)	0.0112 (2)	
O12A	−0.01213 (11)	0.75754 (9)	0.65463 (9)	0.0114 (2)	
O13A	−0.15562 (11)	0.42424 (9)	0.68263 (9)	0.0099 (2)	
H16A	−0.101 (3)	0.395 (2)	0.626 (2)	0.029 (5)*	
C1A	−0.27937 (15)	0.42486 (13)	0.95554 (13)	0.0095 (3)	
C2A	−0.15323 (16)	0.50579 (13)	0.85584 (13)	0.0099 (3)	
H14A	−0.088 (2)	0.5291 (17)	0.9052 (17)	0.011 (4)*	
H15A	−0.209 (2)	0.5897 (19)	0.8023 (18)	0.019 (4)*	
C3A	−0.05408 (15)	0.43861 (13)	0.76326 (13)	0.0087 (3)	
C4A	0.07926 (16)	0.52892 (13)	0.67715 (13)	0.0097 (3)	
H17A	0.138 (2)	0.4872 (18)	0.6159 (18)	0.014 (4)*	
H18A	0.148 (2)	0.5361 (16)	0.7318 (17)	0.010 (4)*	
C5A	0.01286 (15)	0.66847 (13)	0.59661 (13)	0.0088 (3)	
C6A	0.02354 (15)	0.29878 (13)	0.84637 (13)	0.0091 (3)	
H19A	−0.415 (4)	0.402 (3)	1.143 (3)	0.078 (10)*	
H19	−0.090 (4)	0.861 (3)	0.591 (3)	0.069 (9)*	

### (na2c) Disodium hydrogen citrate sesquihydrate Atomic displacement parameters (Å^2^)

**Table:**

	*U*^11^	*U*^22^	*U*^33^	*U*^12^	*U*^13^	*U*^23^
Na1	0.0130 (3)	0.0106 (3)	0.0106 (3)	−0.0013 (2)	−0.0011 (2)	−0.0038 (2)
Na2	0.0106 (3)	0.0124 (3)	0.0117 (3)	−0.0022 (2)	−0.0012 (2)	−0.0053 (2)
Na3	0.0120 (3)	0.0112 (3)	0.0102 (3)	−0.0002 (2)	−0.0019 (2)	−0.0040 (2)
Na4	0.0160 (3)	0.0117 (3)	0.0137 (3)	−0.0042 (2)	−0.0051 (2)	0.0009 (2)
O1W	0.0162 (5)	0.0143 (5)	0.0132 (5)	−0.0023 (4)	−0.0003 (4)	−0.0020 (4)
O2W	0.0163 (5)	0.0145 (5)	0.0150 (5)	−0.0029 (4)	−0.0018 (4)	−0.0021 (4)
O3W	0.0150 (5)	0.0154 (5)	0.0105 (5)	−0.0042 (4)	−0.0025 (4)	−0.0040 (4)
O7	0.0153 (5)	0.0116 (5)	0.0123 (5)	0.0038 (4)	−0.0041 (4)	−0.0064 (4)
O8	0.0144 (5)	0.0127 (5)	0.0108 (5)	0.0010 (4)	−0.0039 (4)	−0.0052 (4)
O9	0.0118 (5)	0.0101 (5)	0.0105 (5)	−0.0037 (4)	−0.0024 (4)	−0.0018 (4)
O10	0.0120 (5)	0.0118 (5)	0.0088 (5)	−0.0023 (4)	−0.0003 (4)	−0.0043 (4)
O11	0.0120 (5)	0.0091 (5)	0.0105 (5)	−0.0018 (4)	−0.0007 (4)	−0.0025 (4)
O12	0.0146 (5)	0.0112 (5)	0.0088 (5)	−0.0031 (4)	−0.0003 (4)	−0.0035 (4)
O13	0.0094 (5)	0.0096 (5)	0.0117 (5)	−0.0024 (4)	−0.0008 (4)	−0.0050 (4)
C1	0.0082 (6)	0.0084 (6)	0.0094 (6)	−0.0032 (5)	0.0011 (5)	−0.0018 (5)
C2	0.0100 (6)	0.0096 (6)	0.0085 (6)	−0.0012 (5)	−0.0018 (5)	−0.0033 (5)
C3	0.0083 (6)	0.0081 (6)	0.0099 (6)	−0.0011 (5)	−0.0017 (5)	−0.0031 (5)
C4	0.0088 (6)	0.0092 (6)	0.0089 (6)	−0.0011 (5)	−0.0006 (5)	−0.0020 (5)
C5	0.0059 (6)	0.0105 (6)	0.0095 (6)	0.0019 (5)	−0.0025 (5)	−0.0025 (5)
C6	0.0064 (6)	0.0077 (6)	0.0103 (7)	0.0026 (5)	−0.0020 (5)	−0.0021 (5)
O7A	0.0178 (5)	0.0129 (5)	0.0108 (5)	−0.0048 (4)	0.0044 (4)	−0.0063 (4)
O8A	0.0139 (5)	0.0090 (5)	0.0102 (5)	−0.0024 (4)	−0.0004 (4)	−0.0029 (4)
O9A	0.0122 (5)	0.0140 (5)	0.0111 (5)	−0.0017 (4)	−0.0043 (4)	−0.0027 (4)
O10A	0.0128 (5)	0.0098 (5)	0.0136 (5)	−0.0006 (4)	−0.0014 (4)	−0.0051 (4)
O11A	0.0126 (5)	0.0116 (5)	0.0085 (5)	−0.0005 (4)	−0.0013 (4)	−0.0026 (4)
O12A	0.0148 (5)	0.0090 (5)	0.0111 (5)	−0.0003 (4)	−0.0034 (4)	−0.0037 (4)
O13A	0.0096 (5)	0.0124 (5)	0.0090 (5)	−0.0007 (4)	−0.0022 (4)	−0.0049 (4)
C1A	0.0101 (6)	0.0088 (6)	0.0083 (6)	0.0018 (5)	−0.0032 (5)	−0.0017 (5)
C2A	0.0105 (6)	0.0097 (6)	0.0094 (6)	−0.0016 (5)	−0.0003 (5)	−0.0035 (5)
C3A	0.0085 (6)	0.0090 (6)	0.0088 (6)	−0.0012 (5)	−0.0022 (5)	−0.0029 (5)
C4A	0.0089 (6)	0.0096 (6)	0.0098 (6)	−0.0004 (5)	−0.0010 (5)	−0.0027 (5)
C5A	0.0053 (6)	0.0101 (6)	0.0102 (6)	−0.0033 (5)	0.0019 (5)	−0.0029 (5)
C6A	0.0063 (6)	0.0111 (6)	0.0077 (6)	−0.0012 (5)	0.0024 (5)	−0.0021 (5)

### (na2c) Disodium hydrogen citrate sesquihydrate Geometric parameters (Å, º)

**Table:**

Na1—O2W^i^	2.3901 (11)	O10—Na2^ix^	2.4085 (10)
Na1—O10^ii^	2.3618 (11)	O10—C6	1.2612 (17)
Na1—O11^iii^	2.4017 (10)	O11—Na1^x^	2.4017 (10)
Na1—O7A	2.3207 (11)	O11—C5	1.2351 (17)
Na1—O8A^iv^	2.7499 (11)	O12—Na4^vii^	2.7435 (11)
Na1—O9A^v^	2.3405 (11)	O12—C5	1.3027 (17)
Na2—O1W^v^	2.4765 (11)	O13—Na2^ix^	2.5209 (11)
Na2—O8^vi^	2.3917 (11)	O13—H16	0.83 (2)
Na2—O10^vi^	2.4085 (10)	O13—C3	1.4158 (16)
Na2—O13^vi^	2.5209 (11)	C1—C2	1.5127 (18)
Na2—O8A	2.4128 (11)	C2—H14	0.940 (18)
Na2—O10A	2.3995 (11)	C2—H15	0.982 (19)
Na2—O13A	2.4647 (11)	C2—C3	1.5282 (18)
Na3—O3W	2.4713 (11)	C3—C4	1.5533 (18)
Na3—O7^vii^	2.3774 (11)	C3—C6	1.5572 (18)
Na3—O8	2.5820 (11)	C4—H17	0.997 (18)
Na3—O9^viii^	2.3989 (10)	C4—H18	0.962 (17)
Na3—O10A^ix^	2.2918 (11)	C4—C5	1.5139 (18)
Na3—O11A^vii^	2.4512 (10)	O7A—C1A	1.2896 (17)
Na4—O1W	2.4397 (12)	O7A—H19A	1.20 (3)
Na4—O2W	2.3810 (12)	O8A—Na1^iv^	2.7499 (11)
Na4—O3W	2.3430 (11)	O8A—Na4^v^	2.3157 (10)
Na4—O9^viii^	2.2851 (10)	O8A—C1A	1.2368 (17)
Na4—O12^vii^	2.7435 (11)	O9A—Na1^v^	2.3405 (11)
Na4—O8A^v^	2.3157 (10)	O9A—C6A	1.2588 (17)
O1W—Na2^v^	2.4765 (11)	O10A—Na3^vi^	2.2918 (11)
O1W—H1W	0.87 (3)	O10A—C6A	1.2528 (17)
O1W—H2W	0.81 (3)	O11A—Na3^vii^	2.4512 (10)
O2W—Na1^viii^	2.3902 (11)	O11A—C5A	1.2342 (17)
O2W—H3W	0.80 (3)	O12A—C5A	1.2997 (17)
O2W—H4W	0.84 (3)	O12A—H19	1.25 (3)
O3W—H5W	0.82 (3)	O13A—H16A	0.84 (2)
O3W—H6W	0.86 (2)	O13A—C3A	1.4157 (16)
O7—Na3^vii^	2.3773 (11)	C1A—C2A	1.5112 (18)
O7—C1	1.2955 (17)	C2A—H14A	0.960 (18)
O7—H19	1.20 (3)	C2A—H15A	0.99 (2)
O8—Na2^ix^	2.3917 (11)	C2A—C3A	1.5268 (18)
O8—C1	1.2357 (17)	C3A—C4A	1.5530 (18)
O9—Na3^i^	2.3988 (10)	C3A—C6A	1.5626 (18)
O9—Na4^i^	2.2851 (10)	C4A—H17A	0.971 (19)
O9—C6	1.2549 (17)	C4A—H18A	0.946 (18)
O10—Na1^ii^	2.3618 (11)	C4A—C5A	1.5156 (18)
			
O2W^i^—Na1—O11^iii^	156.26 (4)	C5—O11—Na1^x^	131.75 (9)
O2W^i^—Na1—O8A^iv^	75.81 (4)	C5—O12—Na4^vii^	152.04 (8)
O10^ii^—Na1—O2W^i^	98.54 (4)	Na2^ix^—O13—H16	90.8 (14)
O10^ii^—Na1—O11^iii^	83.03 (4)	C3—O13—Na2^ix^	106.96 (7)
O10^ii^—Na1—O8A^iv^	87.71 (3)	C3—O13—H16	108.1 (14)
O11^iii^—Na1—O8A^iv^	80.60 (3)	O7—C1—C2	112.53 (11)
O7A—Na1—O2W^i^	97.21 (4)	O8—C1—O7	123.49 (12)
O7A—Na1—O10^ii^	159.74 (4)	O8—C1—C2	123.98 (12)
O7A—Na1—O11^iii^	77.44 (4)	C1—C2—H14	105.8 (10)
O7A—Na1—O8A^iv^	83.87 (4)	C1—C2—H15	104.2 (11)
O7A—Na1—O9A^v^	95.46 (4)	C1—C2—C3	117.38 (11)
O9A^v^—Na1—O2W^i^	88.70 (4)	H14—C2—H15	107.8 (14)
O9A^v^—Na1—O10^ii^	97.56 (4)	C3—C2—H14	111.4 (10)
O9A^v^—Na1—O11^iii^	114.69 (4)	C3—C2—H15	109.7 (10)
O9A^v^—Na1—O8A^iv^	164.25 (4)	O13—C3—C2	107.07 (10)
O8^vi^—Na2—O1W^v^	87.53 (4)	O13—C3—C4	111.32 (10)
O8^vi^—Na2—O10^vi^	86.15 (4)	O13—C3—C6	111.84 (11)
O8^vi^—Na2—O13^vi^	73.84 (4)	C2—C3—C4	109.53 (11)
O8^vi^—Na2—O8A	169.75 (4)	C2—C3—C6	110.59 (10)
O8^vi^—Na2—O10A	91.46 (4)	C4—C3—C6	106.52 (10)
O8^vi^—Na2—O13A	114.69 (4)	C3—C4—H17	109.6 (10)
O10^vi^—Na2—O1W^v^	88.39 (4)	C3—C4—H18	109.9 (9)
O10^vi^—Na2—O13^vi^	66.13 (3)	H17—C4—H18	109.3 (14)
O10^vi^—Na2—O8A	94.93 (4)	C5—C4—C3	111.88 (11)
O10^vi^—Na2—O13A	122.23 (4)	C5—C4—H17	106.8 (10)
O8A—Na2—O1W^v^	82.32 (4)	C5—C4—H18	109.3 (9)
O8A—Na2—O13^vi^	115.91 (4)	O11—C5—O12	122.67 (12)
O8A—Na2—O13A	73.28 (3)	O11—C5—C4	121.45 (12)
O10A—Na2—O1W^v^	81.90 (4)	O12—C5—C4	115.87 (11)
O10A—Na2—O10^vi^	170.09 (4)	O9—C6—O10	125.63 (12)
O10A—Na2—O13^vi^	122.36 (4)	O9—C6—C3	116.04 (11)
O10A—Na2—O8A	85.75 (4)	O10—C6—C3	118.32 (11)
O10A—Na2—O13A	67.43 (3)	C1A—O7A—Na1	142.00 (9)
O13A—Na2—O1W^v^	141.65 (4)	C1A—O7A—H19A	117.2 (15)
O13A—Na2—O13^vi^	69.45 (3)	Na2—O8A—Na1^iv^	83.21 (3)
O3W—Na3—O8	82.77 (3)	Na4^v^—O8A—Na1^iv^	94.23 (3)
O7^vii^—Na3—O3W	98.24 (4)	Na4^v^—O8A—Na2	98.57 (4)
O7^vii^—Na3—O8	91.42 (4)	C1A—O8A—Na1^iv^	120.62 (8)
O7^vii^—Na3—O9^viii^	84.83 (4)	C1A—O8A—Na2	135.06 (9)
O7^vii^—Na3—O11A^vii^	77.67 (4)	C1A—O8A—Na4^v^	115.13 (8)
O9^viii^—Na3—O3W	86.33 (4)	C6A—O9A—Na1^v^	127.26 (9)
O9^viii^—Na3—O8	167.85 (4)	Na3^vi^—O10A—Na2	91.98 (4)
O9^viii^—Na3—O11A^vii^	108.24 (4)	C6A—O10A—Na2	109.60 (8)
O10A^ix^—Na3—O3W	101.16 (4)	C6A—O10A—Na3^vi^	141.08 (9)
O10A^ix^—Na3—O7^vii^	160.52 (4)	C5A—O11A—Na3^vii^	128.77 (9)
O10A^ix^—Na3—O8	89.27 (4)	C5A—O12A—H19	111.2 (13)
O10A^ix^—Na3—O9^viii^	98.11 (4)	Na2—O13A—H16A	90.1 (14)
O10A^ix^—Na3—O11A^vii^	83.15 (4)	C3A—O13A—Na2	108.01 (7)
O11A^vii^—Na3—O3W	164.23 (4)	C3A—O13A—H16A	108.3 (15)
O11A^vii^—Na3—O8	82.12 (3)	O7A—C1A—Na4^v^	90.36 (8)
O1W—Na4—O12^vii^	163.29 (4)	O7A—C1A—C2A	112.97 (11)
O2W—Na4—O1W	99.15 (4)	O8A—C1A—O7A	123.45 (12)
O2W—Na4—O12^vii^	68.41 (4)	O8A—C1A—C2A	123.57 (12)
O3W—Na4—O1W	94.02 (4)	C2A—C1A—Na4^v^	143.09 (9)
O3W—Na4—O2W	165.55 (5)	C1A—C2A—H14A	106.4 (10)
O3W—Na4—O12^vii^	97.48 (4)	C1A—C2A—H15A	105.5 (11)
O9^viii^—Na4—O1W	84.11 (4)	C1A—C2A—C3A	116.50 (11)
O9^viii^—Na4—O2W	95.14 (4)	H14A—C2A—H15A	108.4 (15)
O9^viii^—Na4—O3W	92.09 (4)	C3A—C2A—H14A	111.1 (10)
O9^viii^—Na4—O12^vii^	107.45 (4)	C3A—C2A—H15A	108.6 (11)
O9^viii^—Na4—O8A^v^	169.12 (4)	O13A—C3A—C2A	107.23 (10)
O8A^v^—Na4—O1W	85.14 (4)	O13A—C3A—C4A	110.39 (10)
O8A^v^—Na4—O2W	84.91 (4)	O13A—C3A—C6A	111.81 (10)
O8A^v^—Na4—O3W	90.34 (4)	C2A—C3A—C4A	109.74 (11)
O8A^v^—Na4—O12^vii^	82.72 (3)	C2A—C3A—C6A	109.50 (11)
Na4—O1W—Na2^v^	93.62 (4)	C4A—C3A—C6A	108.16 (10)
Na4—O2W—Na1^viii^	102.60 (4)	C3A—C4A—H17A	108.9 (10)
Na4—O3W—Na3	88.36 (4)	C3A—C4A—H18A	109.7 (10)
C1—O7—Na3^vii^	144.02 (9)	H17A—C4A—H18A	109.0 (14)
C1—O7—H19	116.4 (14)	C5A—C4A—C3A	111.23 (11)
Na2^ix^—O8—Na3	85.37 (3)	C5A—C4A—H17A	107.7 (10)
C1—O8—Na2^ix^	135.12 (9)	C5A—C4A—H18A	110.2 (10)
C1—O8—Na3	116.99 (8)	O11A—C5A—O12A	122.21 (12)
Na4^i^—O9—Na3^i^	91.51 (4)	O11A—C5A—C4A	122.11 (12)
C6—O9—Na3^i^	129.89 (8)	O12A—C5A—C4A	115.66 (12)
C6—O9—Na4^i^	119.94 (8)	O9A—C6A—C3A	116.26 (11)
Na1^ii^—O10—Na2^ix^	92.19 (4)	O10A—C6A—O9A	125.12 (12)
C6—O10—Na1^ii^	140.73 (8)	O10A—C6A—C3A	118.60 (12)
C6—O10—Na2^ix^	110.53 (8)		
			
Na1^iv^—Na2—O8A—Na4^v^	93.28 (4)	O8^vi^—Na2—O10A—C6A	158.19 (9)
Na1^iv^—Na2—O8A—C1A	−127.35 (13)	O8^vi^—Na2—O13A—C3A	−117.77 (8)
Na1^iv^—Na2—O10A—Na3^vi^	−178.42 (3)	O8—Na3—O3W—Na4	−175.84 (4)
Na1^iv^—Na2—O10A—C6A	−31.42 (10)	O8—C1—C2—C3	−11.14 (19)
Na1^iv^—Na2—O13A—C3A	103.13 (8)	O9^viii^—Na3—O3W—Na4	9.53 (4)
Na1^viii^—Na4—O1W—Na2^v^	−43.16 (3)	O9^viii^—Na3—O8—Na2^ix^	138.10 (17)
Na1^viii^—Na4—O3W—Na3	161.99 (4)	O9^viii^—Na3—O8—C1	−82.7 (2)
Na1^ii^—O10—C6—Na2^ix^	121.50 (14)	O9^viii^—Na4—O1W—Na2^v^	−173.86 (4)
Na1^ii^—O10—C6—Na4^i^	−123.34 (11)	O9^viii^—Na4—O2W—Na1^viii^	155.76 (5)
Na1^ii^—O10—C6—O9	−99.84 (16)	O9^viii^—Na4—O3W—Na3	−9.99 (4)
Na1^ii^—O10—C6—C3	79.80 (16)	O10^ii^—Na1—O7A—C1A	165.62 (14)
Na1^x^—O11—C5—O12	8.17 (19)	O10^vi^—Na2—O8A—Na1^iv^	−10.14 (3)
Na1^x^—O11—C5—C4	−173.27 (8)	O10^vi^—Na2—O8A—Na4^v^	83.14 (4)
Na1—O7A—C1A—Na4^v^	177.29 (11)	O10^vi^—Na2—O8A—C1A	−137.49 (12)
Na1—O7A—C1A—O8A	−152.86 (11)	O10^vi^—Na2—O13A—C3A	140.54 (7)
Na1—O7A—C1A—C2A	26.7 (2)	O11^iii^—Na1—O7A—C1A	−178.65 (15)
Na1^iv^—O8A—C1A—Na4^v^	111.85 (10)	O12^vii^—Na4—O1W—Na2^v^	−39.06 (15)
Na1^iv^—O8A—C1A—O7A	65.38 (15)	O12^vii^—Na4—O2W—Na1^viii^	−97.49 (5)
Na1^iv^—O8A—C1A—C2A	−114.13 (12)	O12^vii^—Na4—O3W—Na3	−117.89 (4)
Na1^v^—O9A—C6A—O10A	91.30 (15)	O13^vi^—Na2—O8A—Na1^iv^	55.68 (4)
Na1^v^—O9A—C6A—C3A	−90.26 (13)	O13^vi^—Na2—O8A—Na4^v^	148.96 (4)
Na2^iv^—Na1—O7A—C1A	122.96 (14)	O13^vi^—Na2—O8A—C1A	−71.67 (13)
Na2^ix^—Na3—O3W—Na4	−134.10 (3)	O13^vi^—Na2—O10A—Na3^vi^	−60.54 (5)
Na2^ix^—Na3—O8—C1	139.16 (10)	O13^vi^—Na2—O10A—C6A	86.46 (9)
Na2^v^—Na4—O2W—Na1^viii^	28.21 (4)	O13^vi^—Na2—O13A—C3A	−177.81 (8)
Na2^v^—Na4—O3W—Na3	117.97 (3)	O13—C3—C4—C5	62.49 (14)
Na2^ix^—O8—C1—O7	172.37 (9)	O13—C3—C6—Na2^ix^	−25.63 (9)
Na2^ix^—O8—C1—C2	−7.3 (2)	O13—C3—C6—Na4^i^	−136.59 (11)
Na2^ix^—O10—C6—Na4^i^	115.17 (5)	O13—C3—C6—O9	−176.47 (11)
Na2^ix^—O10—C6—O9	138.67 (11)	O13—C3—C6—O10	3.86 (16)
Na2^ix^—O10—C6—C3	−41.70 (13)	C1—C2—C3—O13	64.88 (14)
Na2^ix^—O13—C3—C2	−88.15 (10)	C1—C2—C3—C4	−174.29 (11)
Na2^ix^—O13—C3—C4	152.17 (8)	C1—C2—C3—C6	−57.19 (15)
Na2^ix^—O13—C3—C6	33.14 (11)	C2—C3—C4—C5	−55.72 (14)
Na2—O8A—C1A—Na4^v^	−134.67 (14)	C2—C3—C6—Na2^ix^	93.60 (9)
Na2—O8A—C1A—O7A	178.86 (9)	C2—C3—C6—Na4^i^	−17.36 (18)
Na2—O8A—C1A—C2A	−0.6 (2)	C2—C3—C6—O9	−57.24 (15)
Na2—O10A—C6A—O9A	136.65 (11)	C2—C3—C6—O10	123.09 (12)
Na2—O10A—C6A—C3A	−41.76 (13)	C3—C4—C5—O11	−85.63 (15)
Na2—O13A—C3A—C2A	−89.61 (10)	C3—C4—C5—O12	93.01 (14)
Na2—O13A—C3A—C4A	150.89 (8)	C4—C3—C6—Na2^ix^	−147.46 (9)
Na2—O13A—C3A—C6A	30.44 (11)	C4—C3—C6—Na4^i^	101.58 (14)
Na3^vi^—Na2—O8A—Na1^iv^	178.64 (3)	C4—C3—C6—O9	61.70 (14)
Na3^vi^—Na2—O8A—Na4^v^	−88.08 (4)	C4—C3—C6—O10	−117.96 (12)
Na3^vi^—Na2—O8A—C1A	51.29 (13)	C6^vi^—Na2—O8A—Na1^iv^	−1.39 (4)
Na3^vi^—Na2—O10A—C6A	147.00 (10)	C6^vi^—Na2—O8A—Na4^v^	91.88 (4)
Na3^vi^—Na2—O13A—C3A	−78.33 (8)	C6^vi^—Na2—O8A—C1A	−128.75 (12)
Na3^vii^—O7—C1—O8	−148.29 (11)	C6^vi^—Na2—O10A—Na3^vi^	4.19 (12)
Na3^vii^—O7—C1—C2	31.4 (2)	C6^vi^—Na2—O10A—C6A	151.19 (11)
Na3—O8—C1—O7	59.85 (15)	C6^vi^—Na2—O13A—C3A	166.14 (7)
Na3—O8—C1—C2	−119.78 (11)	C6^viii^—Na4—O1W—Na2^v^	−156.30 (5)
Na3^i^—O9—C6—Na2^ix^	146.76 (11)	C6^viii^—Na4—O2W—Na1^viii^	141.70 (5)
Na3^i^—O9—C6—Na4^i^	121.40 (13)	C6^viii^—Na4—O3W—Na3	0.92 (5)
Na3^i^—O9—C6—O10	82.71 (16)	C6—C3—C4—C5	−175.35 (11)
Na3^i^—O9—C6—C3	−96.93 (12)	O7A—C1A—C2A—C3A	164.94 (11)
Na3^vi^—O10A—C6A—O9A	−103.39 (16)	O8A^iv^—Na1—O7A—C1A	99.65 (15)
Na3^vi^—O10A—C6A—C3A	78.20 (17)	O8A—Na2—O10A—Na3^vi^	−178.68 (4)
Na3^vii^—O11A—C5A—O12A	12.16 (18)	O8A—Na2—O10A—C6A	−31.68 (9)
Na3^vii^—O11A—C5A—C4A	−169.68 (9)	O8A—Na2—O13A—C3A	55.33 (8)
Na4^i^—Na1—O7A—C1A	63.79 (15)	O8A^v^—Na4—O1W—Na2^v^	4.45 (4)
Na4^v^—Na2—O8A—Na1^iv^	−93.28 (4)	O8A^v^—Na4—O2W—Na1^viii^	−13.32 (5)
Na4^v^—Na2—O8A—C1A	139.37 (14)	O8A^v^—Na4—O3W—Na3	159.40 (4)
Na4^v^—Na2—O10A—Na3^vi^	141.48 (3)	O8A—C1A—C2A—C3A	−15.50 (19)
Na4^v^—Na2—O10A—C6A	−71.52 (8)	O9A^v^—Na1—O7A—C1A	−64.53 (15)
Na4^v^—Na2—O13A—C3A	38.47 (8)	O10A—Na2—O8A—Na1^iv^	179.78 (3)
Na4^i^—O9—C6—Na2^ix^	25.4 (2)	O10A—Na2—O8A—Na4^v^	−86.94 (4)
Na4^i^—O9—C6—O10	−38.70 (17)	O10A—Na2—O8A—C1A	52.43 (12)
Na4^i^—O9—C6—C3	141.66 (9)	O10A—Na2—O13A—C3A	−37.02 (7)
Na4^vii^—O12—C5—O11	105.06 (18)	O10A^ix^—Na3—O3W—Na4	−88.02 (4)
Na4^vii^—O12—C5—C4	−73.6 (2)	O10A^ix^—Na3—O8—Na2^ix^	10.42 (4)
Na4^v^—O8A—C1A—O7A	−46.47 (16)	O10A^ix^—Na3—O8—C1	149.57 (9)
Na4^v^—O8A—C1A—C2A	134.02 (11)	O11A^vii^—Na3—O3W—Na4	167.43 (13)
Na4^v^—C1A—C2A—C3A	39.8 (2)	O11A^vii^—Na3—O8—Na2^ix^	−72.77 (3)
O1W^v^—Na2—O8A—Na1^iv^	−97.84 (4)	O11A^vii^—Na3—O8—C1	66.39 (9)
O1W^v^—Na2—O8A—Na4^v^	−4.56 (4)	O13A—Na2—O8A—Na1^iv^	112.08 (3)
O1W^v^—Na2—O8A—C1A	134.81 (12)	O13A—Na2—O8A—Na4^v^	−154.64 (4)
O1W^v^—Na2—O10A—Na3^vi^	98.48 (4)	O13A—Na2—O8A—C1A	−15.27 (12)
O1W^v^—Na2—O10A—C6A	−114.52 (9)	O13A—Na2—O10A—Na3^vi^	−105.03 (4)
O1W^v^—Na2—O13A—C3A	2.52 (11)	O13A—Na2—O10A—C6A	41.97 (8)
O1W—Na4—O2W—Na1^viii^	70.91 (5)	O13A—C3A—C4A—C5A	59.20 (14)
O1W—Na4—O3W—Na3	74.25 (4)	O13A—C3A—C6A—O9A	−171.79 (11)
O2W^i^—Na1—O7A—C1A	24.84 (15)	O13A—C3A—C6A—O10A	6.76 (16)
O2W—Na4—O1W—Na2^v^	−79.59 (4)	C1A^v^—Na4—O1W—Na2^v^	16.94 (5)
O2W—Na4—O3W—Na3	−130.03 (17)	C1A^v^—Na4—O2W—Na1^viii^	−32.71 (5)
O3W—Na3—O8—Na2^ix^	111.76 (4)	C1A^v^—Na4—O3W—Na3	176.38 (4)
O3W—Na3—O8—C1	−109.08 (10)	C1A—C2A—C3A—O13A	65.52 (14)
O3W—Na4—O1W—Na2^v^	94.45 (4)	C1A—C2A—C3A—C4A	−174.55 (11)
O3W—Na4—O2W—Na1^viii^	−84.54 (18)	C1A—C2A—C3A—C6A	−55.98 (15)
O7^vii^—Na3—O3W—Na4	93.76 (4)	C2A—C3A—C4A—C5A	−58.78 (14)
O7^vii^—Na3—O8—Na2^ix^	−150.11 (4)	C2A—C3A—C6A—O9A	−53.09 (15)
O7^vii^—Na3—O8—C1	−10.96 (9)	C2A—C3A—C6A—O10A	125.46 (12)
O7—C1—C2—C3	169.19 (11)	C3A—C4A—C5A—O11A	−90.55 (15)
O8^vi^—Na2—O8A—Na1^iv^	−105.8 (2)	C3A—C4A—C5A—O12A	87.73 (14)
O8^vi^—Na2—O8A—Na4^v^	−12.5 (2)	C4A—C3A—C6A—O9A	66.46 (14)
O8^vi^—Na2—O8A—C1A	126.9 (2)	C4A—C3A—C6A—O10A	−114.99 (13)
O8^vi^—Na2—O10A—Na3^vi^	11.19 (4)	C6A—C3A—C4A—C5A	−178.18 (11)

Symmetry codes: (i) *x*−1, *y*, *z*; (ii) −*x*−1, −*y*+2, −*z*+2; (iii) *x*, *y*−1, *z*+1; (iv) −*x*−1, −*y*+1, −*z*+2; (v) −*x*, −*y*+1, −*z*+2; (vi) *x*, *y*−1, *z*; (vii) −*x*, −*y*+2, −*z*+1; (viii) *x*+1, *y*, *z*; (ix) *x*, *y*+1, *z*; (x) *x*, *y*+1, *z*−1.

### (na2c_DFT) Crystal data

**Table:**

C_12_H_12_Na_4_O_14_(H_2_O)_3_	α = 68.4610°
*M**_r_* = 526.22	β = 79.6170°
Triclinic, *P*1	γ = 81.7990°
*a* = 8.6713 Å	*V* = 925.63 Å^3^
*b* = 10.6475 Å	*Z* = 2
*c* = 10.9961 Å	*T* = 100 K

### (na2c_DFT) Data collection

**Table:**

*h* = →	*l* = →
*k* = →	

### (na2c_DFT) Fractional atomic coordinates and isotropic or equivalent isotropic displacement parameters (Å^2^)

**Table:**

	*x*	*y*	*z*	*U*_iso_*/*U*_eq_	
Na1	−0.37343	0.67657	1.09352	0.01145*	
Na2	−0.26397	0.20115	0.79858	0.01118*	
Na3	0.08732	1.08255	0.68250	0.01112*	
Na4	0.34457	0.86390	0.85431	0.01460*	
O7	−0.16782	0.95531	0.52502	0.01270*	
O8	−0.20053	1.04584	0.68290	0.01230*	
O9	−0.64112	1.06940	0.68737	0.01090*	
O10	−0.53712	1.16842	0.79941	0.01070*	
O11	−0.47290	1.51265	0.29363	0.01080*	
O12	−0.49766	1.32881	0.24827	0.01140*	
O13	−0.36072	1.31975	0.58091	0.00980*	
H16	−0.42399	1.38378	0.62003	0.02300*	
C1	−0.23465	1.04470	0.57867	0.00910*	
C2	−0.35390	1.14280	0.49792	0.00920*	
H14	−0.42355	1.08420	0.46821	0.00900*	
H15	−0.28636	1.20526	0.40698	0.01500*	
C3	−0.46003	1.23681	0.56155	0.00870*	
C4	−0.58433	1.32299	0.47061	0.00940*	
H17	−0.64219	1.39939	0.51186	0.01100*	
H18	−0.67129	1.25721	0.47345	0.00600*	
C5	−0.51420	1.39486	0.32859	0.00900*	
C6	−0.55479	1.15096	0.69442	0.00870*	
O7A	−0.33595	0.47056	1.05018	0.01370*	
O8A	−0.32484	0.31834	0.95223	0.01120*	
O9A	0.10266	0.30045	0.93971	0.01250*	
O10A	0.00830	0.19041	0.83347	0.01180*	
O11A	−0.01570	0.69714	0.48311	0.01120*	
O12A	−0.01724	0.75439	0.65877	0.01140*	
O13A	−0.15225	0.41596	0.68785	0.00990*	
H16A	−0.08773	0.38671	0.61789	0.02900*	
C1A	−0.27827	0.42098	0.95692	0.00950*	
C2A	−0.15358	0.50329	0.85782	0.00990*	
H14A	−0.08261	0.53524	0.91194	0.01100*	
H15A	−0.21626	0.59489	0.79720	0.01900*	
C3A	−0.05159	0.43504	0.76628	0.00870*	
C4A	0.07944	0.52678	0.67668	0.00970*	
H17A	0.14347	0.47918	0.60764	0.01400*	
H18A	0.16142	0.53410	0.73772	0.01000*	
C5A	0.01259	0.66803	0.59841	0.00880*	
C6A	0.02761	0.29683	0.85249	0.00910*	
H19A	−0.41226	0.40666	1.13119	0.07800*	
H19	−0.09903	0.87170	0.58711	0.06900*	
O1W	0.25806	0.99725	0.99158	0.01550*	
H1W	0.34046	1.05682	0.93555	0.04700*	
H2W	0.16299	1.05211	0.95978	0.03700*	
O2W	0.61368	0.82806	0.87514	0.01610*	
H3W	0.68826	0.89557	0.85395	0.04700*	
H4W	0.64281	0.78276	0.81112	0.06700*	
O3W	0.08969	0.85012	0.82260	0.01340*	
H5W	0.05984	0.80182	0.77073	0.03600*	
H6W	0.01544	0.82092	0.90413	0.02900*	

### (na2c_DFT) Bond lengths (Å)

**Table:**

Na1—O10^i^	2.333	C1—C2	1.513
Na1—O11^ii^	2.357	C2—H14	1.090
Na1—O7A	2.377	C2—H15	1.094
Na1—O8A^iii^	2.741	C2—C3	1.536
Na1—O9A^iv^	2.344	C3—C4	1.561
Na1—O2W^v^	2.363	C3—C6	1.564
Na2—O8^vi^	2.385	C4—H17	1.092
Na2—O10^vi^	2.441	C4—H18	1.089
Na2—O13^vi^	2.496	C4—C5	1.519
Na2—O8A	2.393	O7A—C1A	1.311
Na2—O10A	2.440	O7A—H19A	1.079
Na2—O13A	2.411	O8A—Na1^iii^	2.741
Na2—O1W^iv^	2.494	O8A—Na4^iv^	2.292
Na3—O7^vii^	2.424	O8A—C1A	1.239
Na3—O8	2.580	O9A—Na1^iv^	2.344
Na3—O9^viii^	2.348	O9A—C6A	1.267
Na3—O10A^ix^	2.304	O10A—Na3^vi^	2.304
Na3—O11A^vii^	2.462	O10A—C6A	1.263
Na3—O3W	2.387	O11A—Na3^vii^	2.462
Na4—O9^viii^	2.284	O11A—C5A	1.251
Na4—O8A^iv^	2.292	O12A—C5A	1.293
Na4—O1W	2.391	O13A—H16A	0.986
Na4—O2W	2.349	O13A—C3A	1.413
Na4—O3W	2.331	C1A—C2A	1.506
O7—Na3^vii^	2.424	C2A—H14A	1.090
O7—C1	1.312	C2A—H15A	1.092
O7—H19	1.079	C2A—C3A	1.535
O8—Na2^ix^	2.385	C3A—C4A	1.556
O8—C1	1.239	C3A—C6A	1.568
O9—Na4^v^	2.284	C4A—H17A	1.092
O9—Na3^v^	2.348	C4A—H18A	1.090
O9—C6	1.255	C4A—C5A	1.523
O10—Na2^ix^	2.441	O1W—Na2^iv^	2.494
O10—Na1^i^	2.333	O1W—H1W	0.988
O10—C6	1.273	O1W—H2W	0.980
O11—Na1^x^	2.357	O2W—Na1^viii^	2.363
O11—C5	1.254	O2W—H3W	0.972
O12—C5	1.295	O2W—H4W	0.971
O13—Na2^ix^	2.496	O3W—H5W	0.981
O13—H16	0.987	O3W—H6W	0.979
O13—C3	1.410		

Symmetry codes: (i) −*x*−1, −*y*+2, −*z*+2; (ii) *x*, *y*−1, *z*+1; (iii) −*x*−1, −*y*+1, −*z*+2; (iv) −*x*, −*y*+1, −*z*+2; (v) *x*−1, *y*, *z*; (vi) *x*, *y*−1, *z*; (vii) −*x*, −*y*+2, −*z*+1; (viii) *x*+1, *y*, *z*; (ix) *x*, *y*+1, *z*; (x) *x*, *y*+1, *z*−1.

### (na2c_DFT) Hydrogen-bond geometry (Å, º)

**Table:**

*D*—H···*A*	*D*—H	H···*A*	*D*···*A*	*D*—H···*A*
O7*A*—H19*A*···O12	1.079	1.393	2.465	171.1
O7—H19···O12*A*	1.079	1.382	2.456	172.5
O13*A*—H16*A*···O11*A*	0.986	1.725	2.698	168.3
O13—H16···O11	0.987	1.760	2.743	173.4
O1*W*—H1*W*···O10	0.988	1.806	2.772	165.0
O3*W*—H5*W*···O12*A*	0.981	1.751	2.714	165.9
O3*W*—H6*W*···O9*A*	0.979	1.945	2.881	159.0
O1*W*—H2*W*···O10*A*	0.980	2.122	3.067	161.4
O2*W*—H4*W*···O12	0.971	2.171	2.877	128.5
O2*W*—H3*W*···O8	0.972	2.146	2.946	138.6
O2*W*—H3*W*···O1*W*	0.972	2.503	3.166	125.3
